# Aspects of the rhizospheric microbiota and their interactions with the soil ecosystem

**DOI:** 10.18699/VJGB-22-54

**Published:** 2022-08

**Authors:** Belkacem El Amrani

**Affiliations:** Department of Biology, Faculty of Science Dhar El Mehraz, Sidi Mohamed Ben Abdellah University, Fes, Morocco

**Keywords:** soil microorganisms, rhizosphere, microbial diversity, plant biodiversity, почвенные микроорганизмы, ризосфера, микробное разнообразие, биоразнообразие растений

## Abstract

Soil microbial communities play a key role in the evolution of the rhizosphere. In addition, proper exploration of these microbial resources represents a promising strategy that guarantees the health and sustainability of all ecosystems connected to the ground. Under the inf luence of environmental conditions, microbial communities can change compositions in terms of abundance and diversity. Beyond the descriptive level, the current orientation of microbial ecology is to link these structures to the functioning of ecosystems; specif ically, to understand the effect of environmental factors on the functional structure of microbial communities in ecosystems. This review focuses on the main interactions between the indigenous soil microf lora and the major constituents of the rhizosphere to understand, on the one hand, how microbial biodiversity can improve plant growth and maintain homeostasis of the rhizospheric ecosystem, on the other hand, how the maintenance and enrichment of plant biodiversity can contribute to the conservation of soil microbial diversity; knowing that these microorganisms are also controlled by the abiotic properties of the soil. Overall, understanding the dynamics of the rhizosphere microbiome is essential for developing innovative strategies in the f ield of protecting and maintaining the proper functioning of the soil ecosystem

## Abstract (ru)

Почвенные микробные сообщества играют ключевую роль в эволюции ризосферы. Планомерное изучение этих микробных ресурсов представляет собой перспективную стратегию, с помощью которой можно будет обеспечить здоровье и устойчивость всех почвенных экосистем. Под воздействием окружающей среды микробные сообщества могут менять численность своих популяций и видовой состав. Современная микробная экология нацелена, помимо описательного уровня, на определение связей этих структур с функционированием экосистем, в частности для понимания роли окружающей среды в жизнедеятельности микробных сообществ в экосистемах. Настоящий обзор посвящен основным взаимодействиям между местной почвенной микрофлорой и главными составляющими ризосферы. Важно понять, с одной стороны, , как микробное биоразнообразие может улучшить рост растений и поддержать гомеостаз ризосферной экосистемы, а с другой – как сохранение и повышение растительного биоразнообразия способствуют сохранению почвенного микробного разнообразия, зная при этом, что данные микроорганизмы контролируются еще и абиотическими свойствами почв. В целом понимание динамики микробиома ризосферы необходимо для разработки инновационных стратегий в области защиты и поддержания надежного функционирования почвенной экосистемы.

## Introduction

The rhizosphere, a narrow area of soil that surrounds the roots of plants, harbors a number of microorganisms that interact with plants and the surrounding soil, and is considered one of the most dynamic interfaces on Earth (Philippot et al., 2013; Kuzyakov, Blagodatskaya, 2015). In addition, since their colonization of terrestrial environments, terrestrial plants have formed symbioses with microorganisms (Fitzpatrick et al., 2018). They have been accompanied by fungi, bacteria, viruses and protists over millions of years, and those associations that allow and accelerate the adaptation of plants to life on Earth (Shekhar et al., 2019).

It has been estimated that the symbiosis between plants and fungi was established early with arbuscular mycorrhizal fungi more than 450 million years ago (Ma) during the colonization of the Earth by plants, as the oldest and the most common symbiotic association of plants with microbes (Field et al., 2015). However, the structure and activity of soil microbial communities are intimately linked to their roles in ecological processes; the identity and abundance of species present in an ecosystem determine the types of interactions in the rhizosphere and subsequently constitute the key elements of the ecological theories (Talbot et al., 2014). In addition, the soil microbiome is divided into two distinct microbial compartments, depending on their position in relation to the roots of plants, the microorganisms surrounding the roots being commonly referred to as rhizospheric or endophytes (Fitzpatrick et al., 2018).

Interactions between the plant and its microbiota range from parasitism to mutualism, and their results can be decisive for the performance of the plant (Almario et al., 2017; El Amrani, Amraoui, 2022). Endophytic soil microorganisms colonize plant roots forming complex communities and perform beneficial functions by improving plant growth, health and defense against enemies. This association improves the adaptation of plants to environmental constraints such as drought and nutrient deficiency (Almario et al., 2017; Shekhar et al., 2019). This beneficial effect of the root microbiota on plants is achieved by the secretion of different growth hormones such as auxin, cytokinin and gibberellic acid, or by reducing the production of ethylene. This leads to the promotion of plant growth by changing the architecture of the root system (Shekhar et al., 2019; El Amrani, Amraoui, 2020) and also by increasing the acquisition of nutrients (Fitzpatrick et al., 2018).

Thus, the plant microbiota can be considered as an extension of the plant, in the sense that it can increase the plant’s access to nutrients in poor soils (Vandenkoornhuyse et al., 2015). It has been estimated that 80 % of vascular plant species receive phosphorus (P) and other nutrients from fungi in exchange for photosynthesis (Almario et al., 2017) (see the Figure). In other words, microbial biodiversity is essential to enhance the sustainable growth of plants through improved nutrition, root architecture, defense mechanisms and the competition with pathogens as well as through participation in the adaptation of plants to abiotic constraints.

**Fig. 1.**
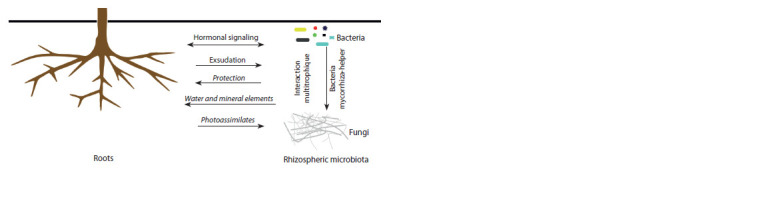
Schematic of the interactions between roots and soil microbial communities

## The concept of microbial biodiversity

Bacteria are the most diverse organisms among living things (Whitman et al., 1998). Their activity, richness and composition play a major role in the functioning of an ecosystem, either free-living or associated with other host organisms (Walters, Martiny, 2020).

Microbial biodiversity studies use several methods depending on the objective. Species diversity is the most studied concept despite it being a single dimension of biodiversity (Latimer, 2012). This is due to the fact that species is the basic unit of ecology and the evolution of ecosystems, hence the importance of this concept for evaluating and conserving biodiversity. However, definitions and formulas have been developed to fully understand and control microbial communities (Fontana et al., 2020). These notions include the measurement of biodiversity at spatial scales; according to Whittaker (1972), this notion is based on three scopes: (i) alpha diversity refers to the diversity within a particular ecosystem (number or relative abundance of taxa); (ii) beta diversity expresses the total number of species unique to each of the ecosystems compared; it makes it possible to examine the evolution of the diversity of species between several ecosystems; (iii) the total or gamma diversity of a landscape, or geographical area, is the product of the alpha diversity of its communities and the degree of beta differentiation between them. Among these three parameters, alpha diversity is the key element in conservation work because it quantifies the biodiversity of a particular ecosystem through measurement based on the notion of presence/absence and abundance of taxa within a local community.

Despite the diversification of these mathematical tools, they fail to reflect the added value of diversity within the ecological whole. In this regard, the notion of functional diversity versus specific diversity appeared (Biswas, Mallik, 2011). This functional diversity is based on a metric for quantifying the diversity of functional traits (Díaz et al., 2007). This calls into question the philosophy of conservation biology, which recognizes that the great diversity of species ensures great functional diversity and maintains the functional stability of the ecosystem (Mayfield et al., 2010).

## Factors and interactions of soil microorganisms

Soil microbial communities are vital for an ecosystem to maintain the sustainability of long-term ecological interactions (Chang et al., 2017). They are essential to the plant due to their contribution to its growth, its development and its productivity (Trivedi et al., 2013) through the maintenance of soil fertility thanks to the important roles they play in the availability of nutrients (Chang et al., 2017). Soil microbial communities also play a fundamental role in soil biogeochemical cycles (Rousk, Bengtson, 2014) because the dynamic structure of soil largely depends on the interaction between microbial biology and the roots of plants living in the soil (Jin K. et al., 2013).

However, there are a variety of factors that can significantly affect soil microbial communities and predict the abundance and diversity of these communities. Among these factors, there are biotic factors such as root respiration and the nature of forest formation (Chen et al., 2015b; Schmid et al., 2019); and abiotic factors such as temperature, climate, soil pH, moisture, organic matter also including nutritive elements such as nitrogen and phosphorus (Cao et al., 2016; Wang et al., 2018; Chernov, Zhelezova, 2020). These biotic and abiotic factors are very dynamic and consist of many elements that can interact and influence microbial communities in the soil. Some studies have elucidated that the interaction between microbial communities and soil biotic and abiotic factors functions as an integrated impact of climate-soil-plant factors on the soil microbiome (Jiménez et al., 2019; Pingel et al., 2019). More so, soil microbial communities react primarily in response to changes in plant-soil interactions (Yao et al., 2018). Therefore, these microbial communities are essential in order to maintain homeostasis of the entire rhizospheric ecosystem (Raaijmakers et al., 2009).

## Afforestation and soil microorganisms

Afforestation has a very important role in the functioning of rhizospheric ecosystems, it improves soil conditions and promotes soil development, especially in degraded ecosystems with an extremely poor environment (Ren et al., 2017; El Amrani, Amraoui, 2018). In addition, soil microbes react quickly to afforestation, which causes a huge increase in microbial proliferation (van der Wal et al., 2006). Dominant bacterial phyla, both Proteobacteria, Bacteroidetes, have been shown to be significantly more abundant in woodland than in abandoned land (Baldrian, 2017; Ren et al., 2018). In addition, the development of fungal communities also shows a significant increase after afforestation (Wallander et al., 2013; Gunina et al., 2017) in response to the improvement of the chemical properties of the soil as in the case of the conversion of abandoned land into forest (Yang et al., 2018).

However, natural forest ecosystems maintain greater soil microbial diversity than monoculture afforestation (Monkai et al., 2018). Some studies have shown that Ascomycota responded positively to land use change while Basidiomycota responded negatively (Ren et al., 2018). Also, ecosystems that contain a mixture of different plant genera have the potential to exhibit greater microbial community heterogeneity than single species plantations (Carnovale et al., 2019). From this proposition, it can be concluded that changes due to afforestation type may be related mainly to fungal phyla. Finally, this shows that maintaining the variability of plant species during afforestation greatly contributes to the conservation of the microbial diversity of the soil.

## Plant species and soil microorganisms

The effect of afforestation on microbial communities may be due to the nature and diversity of plant species. In addition, plant species have been shown to significantly influence the composition and microbial structure of the soil (Yang et al., 2018). Therefore, the structure and function of the soil microbial community are often shown to be spatially associated with the composition, richness and biomass of plant communities (Gömöryová et al., 2013; Carnovale et al., 2019), as well as with stages of plant growth (Sheng et al., 2017). In addition, it has been believed that the soil microbiota responds quickly to variations in plant species (Yang et al., 2018) due to direct interaction between plant roots and soil microorganisms (Gömöryová et al., 2013). But this effect is not homogeneous and it is more pronounced on fungal communities than on bacterial communities (Carnovale et al., 2019). However, in addition to the direct effect of plant species on soil microbial communities, the structure and function of plant communities can indirectly influence (inhibit or stimulate) these microbial communities by altering the physical and chemical properties of the soil (Shen et al., 2013; El Amrani, 2017; Yang et al., 2018). Therefore, plant roots exert a strong impact on soil pathogens and beneficial microorganisms in the rhizosphere by producing exudates as well as secondary metabolites (Feng et al., 2019). Therefore, the enrichment of plant biodiversity plays a vital role in maintaining the microbial composition of the soil, which is not the case with monocultures. This conclusion is confirmed by the works of Schmid et al. (2019) who has tested, over the course of 11 years, soil bacterial communities developed under plant monocultures and mixtures. These works confirm that richness in plant species positively affects the composition and diversity of microbial communities.

Rhizospheric bacterial communities are considered cosmopolitan and colonize all biogeographical regions (Hanson et al., 2012). However, their activities and their diversities as well as their distributions are controlled by several parameters of the environment; among these factors, the plant figures as the major factor that controls them (Kumar et al., 2017). Some research has found conflicting results regarding prediction of microbial diversity by plant diversity when examining their relationships at large spatial scales (Liu et al., 2020). However, microbial communities are composed of groups that differ in their behavior. In this regard, we cite the obligate pathogenic or symbiotic microorganisms, the life cycles of which can only be completed in the presence of their specific host such as obligate endophytes (Sally, David, 2008; Nair, Padmavathy, 2014; Glick, 2020). Another example can be seen in the case of ectomycorrhizae, most of which present host-symbiont specificity (Kernaghan et al., 2003). According to these two examples, we can only admit that parental control exerted by plant diversity influences the activity and microbial diversity of the rhizosphere. However, the degree of this control differs by several parameters mainly including the nature of microbial groups, plant species, and also soil and climatic conditions (Bargali et al., 2018; D’Acunto et al., 2018; Malard, Pearce, 2018). This explains the sometimes modest correlations between microbial richness and plant diversity (Liu et al., 2020).

Bulgarelli et al. (2015) used the term ‘domestication’ of bacterial communities by plant roots to explain the dominance of three bacterial families Comamonadaceae, Flavobacteriaceae and Rhizobiaceae in the barley root microbiota. On the other hand, a broad conservation of the composition of the root bacterial microbiota has been found in Arabidopsis thaliana and related species extending over 35 Ma within the family Brassicaceae (Schlaeppi et al., 2014). These results mean that the host plant determines and maintains its bacterial procession. This control of the soil microbial diversity by the plants is carried out mainly by the process of rhizodeposition (root excretion of photosynthesis-derived organic compounds) (Jones et al., 2009). These exudates can influence this microbial community either through trophic selection (trophic substances used by specific microbial groups) (Mansouri et al., 2002), biochemical selection (substances that stimulate or inhibit the proliferation of a given microbial group) (Rosier et al., 2018) or by chemotaxis (substances that attract targeted microbial groups to the roots of the plant) (Scharf et al., 2016).

## Litter and soil microorganisms

The main methods by which plant communities affect soil chemical properties and subsequently microbial communities are primarily root and leaf litter, and root exudates (Zverev et al., 2016). Trees produce the majority of the waste deposited on the ground, in addition to a very large part of root exudates and dead roots under the ground (Gömöryová et al., 2013), which provides different inputs in quantity and quality (Yao et al., 2018). It is essential to claim that trees influence the soil microbiota basically in the same way as other plants, but their effect is potentially stronger due to the greater input biomass (Gömöryová et al., 2013). Therefore, the difference in the quantity and quality of litter and exudate inputs, different species and plant communities, modulates and causes a change in soil microbial communities (Santonja et al., 2018) even at the regional scale (Chen et al., 2015a).

Likewise, several previous studies have reported that differences in litter quality between tree species affect the abundance and composition of bacterial and fungal communities in the soil (Santonja et al., 2018; Pingel et al., 2019). In addition, differences in the quality of the litter occur in the nature of the inputs; such as the leaching of dissolved organic matter and nutrients, and the exudation of different kinds of ions and organic compounds (Yang et al., 2018). These variations can alter the rate and speed of fundamental soil processes, such as nutrient cycling and carbon dynamics, differently (Carnovale et al., 2019). Consequently, the greatest effect of plant species on the chemical properties of the soil is observed in the topsoil corresponding to the greatest amount of organic matter introduced (Kooch et al., 2017). From these results, it is clear that the quality and quantity of litter entering the soil are a determining factor in the existence of microbial communities and needs to be further investigated.

## Secondary metabolites and soil microorganisms

Secondary metabolites are another component of plant litter of particular interest to soil ecosystems and exert a major effect on their edaphic microflora, especially in forest soils where complex phenol content is significantly higher (Yang et al., 2018). Similarly, Santonja et al. (2018) showed a contrasting activity of bacterial and fungal communities in response to the diversity of plant litter in a Mediterranean forest. These authors and others have shown that secondary metabolites repress biomass and the activity of microbial communities (Chomel et al., 2016; Santonja et al., 2018). Likewise, Chomel et al. (2014) showed a strong inhibitory effect of phenolic compounds, depending on the concentration, on fungal biomass in a Mediterranean pine forest. On the other hand, Amaral and Knowles (1998) reported the presence of monoterpenes negatively affecting the growth and activity of certain soil microbial groups while having a positive effect on other groups. However, knowledge of the effects of secondary metabolites on the activity and richness of soil microbial communities is still very limited.

## Soil pH and microorganism communities

The change in pH is also a consequence of the biogeochemical interaction and has a major effect on the composition and activity of the soil microbial community. Therefore, the pH represents the primary metabolic control of microbial communities (Zhalnina et al., 2015). This control can be direct, by modulating the thermodynamics and kinetics of redox reactions and microbial respiration thereafter; or indirect by determining salinity and nutrient bioavailability through determination of proton chemical activity, mineral dissolution and precipitation, and other geochemical reactions (Bethke et al., 2011). On the other hand, soil pH describes the extracellular enzymatic activities and the rate of decomposition of organic matter (Jin Q., Kirk, 2018).

It has been reported that changes in the composition and diversity of microbial communities are positively correlated with variation in soil pH and that this variation controls their spatial distribution in the rhizosphere (Shen et al., 2013). This distribution was lower in monoculture plantations than in natural forests (Monkai et al., 2018). As reported in the study of Chen et al. (2015b), soil acidification decreased soil microbial respiration in forest ecosystems. These results suggest that reducing soil pH can lead to decreased biodiversity, rates of biogeochemical cycling, and ecosystem functioning (Chen et al., 2015b). Unlike bacterial communities, soil acidification has a slightly favorable effect on the richness of fungi in forest ecosystems (Rousk et al., 2011). Thus, the advanced knowledge of these interactions (pH-fungi-bacteria) can be a very powerful tool to mitigate negative effects caused by pathogenic fungi or bacteria by increasing or decreasing soil acidity.

## Climate and soil microorganisms

The climatic conditions of soil ecosystems constitute one of the most determining parameters of the distribution of microbial communities. Previous research has confirmed that the spatial variation of soil microbial biomass depends on the spatial heterogeneity of climatic conditions (Xu et al., 2018). This justifies the use of microbiological properties as better indicators of soil quality, in particular the great capacity of microbial communities to react quickly to environmental changes (Marinari et al., 2006). As an example, several studies have reported that the mean annual temperature and mean annual precipitation show a positive correlation with microbial abundance and diversity (Cao et al., 2016; Tu et al., 2016). Also, low soil moisture and dry conditions during the summer drought period have a negative effect on microbial diversity and richness. These types of conditions can make a specific selection through the selection of drought resistant taxa such as fungi with lower nutritional requirements and higher water acquisition capacity or Gram positive bacteria (Manzoni et al., 2012; Xi et al., 2018).

From these results and the fact that soils belonging to the same climatic types have similar properties, we can conclude that climatic factors are of great importance for biodiversity and the richness of microbial biomass in the soil. It also suggests that soil microbes could be used as a more precise indicator of soil ecosystem characteristics

## Soil depth and soil microorganisms

Little is known about the effects of the physical properties of soil on the plant-microorganism interaction. However, the physical properties of soil have been reported to cause profound changes in soil microbial communities (Thoms et al., 2010; Xu et al., 2018). In addition to the physical properties of soil, the biomass and activities of fungal and bacterial communities also change at different depths of the soil profile (Carnovale et al., 2019). This vertical distribution reveals that fungi predominate in the topsoil of the soil, generally between 0 and 10 cm deep, and bacteria and actinomycetes predominate deep soils between 40 and 100 cm deep (Yao et al., 2018).

Nevertheless, it remains necessary to understand how physical properties, especially mechanical ones, can influence the microbiome residing in the soil and what mechanisms the microbiome can use to combat these types of stresses.

## Conclusion

Microbial biodiversity is essential for improving sustainable plant growth and maintaining homeostasis of the entire rhizospheric ecosystem. In return, maintaining and enriching plant biodiversity greatly contributes to the conservation of soil microbial diversity. However, this balance depends and/or at the same time affects the biogeochemical cycle of the soil. Taken together, these interactions explain the complexity of understanding the dynamics of the rhizospheric microbiome. Hence the importance of such a study that could inform future work aimed at researching the interactions between microbial communities and other soil components in order to improve the management of resources and the productivity of rhizospheric ecosystems.

## Conflict of interest

The authors declare no conflict of interest.

## References

Almario J., Jeena G., Wunder J., Langen G., Zuccaro A., Coupland G.,
Bucher M. Root-associated fungal microbiota of nonmycorrhizal
Arabis alpina and its contribution to plant phosphorus nutrition.
Proc. Natl. Acad. Sci. USA. 2017;114:E9403-E9412. DOI 10.1073/
pnas.1710455114.

Amaral J.A., Knowles R. Inhibition of methane consumption in forest
soils by monoterpenes. J. Chem. Ecol. 1998;24:723-734. DOI
10.1023/A:1022398404448

Baldrian P. Forest microbiome: diversity, complexity and dynamics.
FEMS Microbiol. Rev. 2017;41(2):109-130. DOI 10.1093/femsre/
fuw040.

Bargali K., Manral V., Padalia K., Bargali S.S., Upadhyay V.P. Effect
of vegetation type and season on microbial biomass carbon in
Central
Himalayan forest soils, India. CATENA. 2018;171:125-135.
DOI 10.1016/j.catena.2018.07.001.

Bethke C.M., Sanford R.A., Kirk M.F., Jin Q., Flynn T.M. The thermodynamic
ladder in geomicrobiology. Am. J. Sci. 2011;311(3):183-
210. DOI 10.2475/03.2011.01.

Biswas S.R., Mallik A.U. Species diversity and functional diversity relationship
varies with disturbance intensity. Ecosphere. 2011;2(4):
1-10. DOI 10.1890/ES10-00206.1.

Bulgarelli D., Garrido-Oter R., Münch P.C., Weiman A., Dröge J.,
Pan Y., McHardy A.C., Schulze-Lefert P. Structure and function of
the bacterial root microbiota in wild and domesticated barley. Cell
Host Microbe. 2015;17(3):392-403. DOI 10.1016/j.chom.2015.
01.011.

Cao H., Chen R., Wang L., Jiang L., Yang F., Zheng S., Wang G., Lin X.
Soil pH, total phosphorus, climate and distance are the major factors
influencing microbial activity at a regional spatial scale. Sci. Rep.
2016;6:25815. DOI 10.1038/srep25815.

Carnovale D., Bissett A., Thrall P.H., Baker G. Plant genus (Acacia and
Eucalyptus) alters soil microbial community structure and relative
abundance within revegetated shelterbelts. Appl. Soil Ecol. 2019;
133:1-11. DOI 10.1016/j.apsoil.2018.09.001

Chang E.-H., Tian G., Chiu C.-Y. Soil microbial communities in natural
and managed cloud montane forests. Forests. 2017;8(2):33. DOI
10.3390/f8010033.

Chen D., Mi J., Chu P., Cheng J., Zhang L., Pan Q., Xie Y., Bai Y. Patterns
and drivers of soil microbial communities along a precipitation
gradient on the Mongolian Plateau. Landsc. Ecol. 2015a;30:1669-
1682. DOI 10.1007/s10980-014-9996-z.

Chen D., Wang Y., Lan Z., Li J., Xing W., Hu S., Bai Y. Biotic community
shifts explain the contrasting responses of microbial and root
respiration to experimental soil acidification. Soil Biol. Biochem.
2015b;90:139-147. DOI 10.1016/j.soilbio.2015.08.009

Chernov T.I., Zhelezova A.D. The dynamics of soil microbial communities
on different timescales: a review. Eurasian Soil Sci. 2020;53:
643-652. DOI 10.1134/S106422932005004X.

Chomel M., Fernandez C., Bousquet-Mélou A., Gers C., Monnier Y.,
Santonja M., Gauquelin T., Gros R., Lecareux C., Baldy V. Secondary
metabolites of Pinus halepensis alter decomposer organisms
and litter decomposition during afforestation of abandoned agricultural
zones. J. Ecol. 2014;102(2):411-424. DOI 10.1111/1365-2745.
12205.

Chomel M., Guittonny-Larchevêque M., Fernandez C., Gallet C.,
DesRochers
A., Paré D., Jackson B.G., Baldy V. Plant secondary
metabolites: a key driver of litter decomposition and soil nutrient
cycling. J. Ecol. 2016;104(6):1527-1541. DOI 10.1111/1365-2745.
12644.

D’Acunto L., Andrade J.F., Poggio S.L., Semmartin M. Diversifying
crop rotation increased metabolic soil diversity and activity of the
microbial community. Agric. Ecosyst. Environ. 2018;257:159-164.
DOI 10.1016/j.agee.2018.02.011.

Díaz S., Lavorel S., de Bello F., Quétier F., Grigulis K., Robson T.M.
Incorporating plant functional diversity effects in ecosystem service
assessments. Proc. Natl. Acad. Sci. USA. 2007;104(52):20684-
20689. DOI 10.1073/pnas.0704716104.

El Amrani B. The effect of pH on the growth of Cedrus atlantica M.
plants. 1st Scientific Day dedicated to PhD students under the theme
“Biotechnology, Ecology and Valorization of Phyto-resources”
FSDM, Fes, Morocco. 2017. DOI 10.5281/zenodo.619302.

El Amrani B., Amraoui B.M. Effects of some properties of cedar forest
soils on secondary roots of Cedrus atlantica Manetti. J. For. Sci.
2018;64:506-513. DOI 10.17221/69/2018-JFS.

El Amrani B., Amraoui B.M. Biomechanics of Atlas cedar roots in response
to the medium hydromechanical characteristics. Scientifica.
2020;2020:7538698. DOI 10.1155/2020/7538698.

El Amrani B., Amraoui B.M. Soil microbial communities affect development
of Cedrus atlantica M. Asian J. Soil Sci. Plant Nutr.
2022;7(1):43-50.

Feng Y., Hu Y., Wu J., Chen J., Yrjälä K., Yu W. Change in microbial
communities, soil enzyme and metabolic activity in a Torreya
grandis plantation in response to root rot disease. For. Ecol. Manag.
2019;432:932-941. DOI 10.1016/j.foreco.2018.10.028.

Field K.J., Leake J.R., Tille S., Allinson K.E., Rimington W.R., Bidartondo
M.I., Beerling D.J., Cameron D.D. From mycoheterotrophy
to mutualism: mycorrhizal specificity and functioning in Ophioglossum
vulgatum sporophytes. New Phytol. 2015;205:1492-1502. DOI
10.1111/nph.13263.

Fitzpatrick C.R., Copeland J., Wang P.W., Guttman D.S., Kotanen P.M.,
Johnson M.T.J. Assembly and ecological function of the root microbiome
across angiosperm plant species. Proc. Natl. Acad. Sci. USA.
2018;115(6):E1157-E1165. DOI 10.1073/pnas.1717617115.

Fontana V., Guariento E., Hilpold A., Niedrist G., Steinwandter M.,
Spitale D., Nascimbene J., Tappeiner U., Seeber J. Species richness
and beta diversity patterns of multiple taxa along an elevational gradient
in pastured grasslands in the European Alps. Sci. Rep. 2020;
10:12516. DOI 10.1038/s41598-020-69569-9.

Glick B.R. Beneficial Plant-Bacterial Interactions. Springer Cham,
2020. DOI 10.1007/978-3-030-44368-9.

Gömöryová E., Ujházy K., Martinák M., Gömöry D. Soil microbial
community response to variation in vegetation and abiotic environment
in a temperate old-growth forest. Appl. Soil Ecol. 2013;68:10-
19. DOI 10.1016/j.apsoil.2013.03.005.

Gunina A., Smith A.R., Godbold D.L., Jones D.L., Kuzyakov Y. Response
of soil microbial community to afforestation with pure and
mixed species. Plant Soil. 2017;412:357-368. DOI 10.1007/s11104-
016-3073-0.

Hanson C.A., Fuhrman J.A., Horner-Devine M.C., Martiny J.B.H.
Beyond biogeographic patterns: processes shaping the microbial
landscape. Nat. Rev. Microbiol. 2012;10:497-506. DOI 10.1038/
nrmicro2795.

Jiménez J.J., Igual J.M., Villar L., Benito-Alonso J.L., Abadias-Ullod J.
Hierarchical drivers of soil microbial community structure variability
in “Monte Perdido” Massif (Central Pyrenees). Sci. Rep. 2019;
9(1):8768. DOI 10.1038/s41598-019-45372-z.

Jin K., Shen J., Ashton R.W., Dodd I.C., Parry M.A.J., Whalley W.R.
How do roots elongate in a structured soil? J. Exp. Bot. 2013;64(15):
4761-4777. DOI 10.1093/jxb/ert286.

Jin Q., Kirk M.F. pH as a primary control in environmental microbiology:
1. Thermodynamic perspective. Front. Environ. Sci. 2018;
6:21. DOI 10.3389/fenvs.2018.00021

Jones D.L., Nguyen C., Finlay R.D. Carbon flow in the rhizosphere:
carbon trading at the soil–root interface. Plant Soil. 2009;321:5-33.
DOI 10.1007/s11104-009-9925-0.

Kernaghan G., Widden P., Bergeron Y., Légaré S., Paré D. Biotic and
abiotic factors affecting ectomycorrhizal diversity in boreal mixedwoods.
Oikos. 2003;102:497-504.

Kooch Y., Samadzadeh B., Hosseini S.M. The effects of broad-leaved
tree species on litter quality and soil properties in a plain forest stand.
CATENA. 2017;150:223-229. DOI 10.1016/j.catena.2016.11.023.

Kumar M., Brader G., Sessitsch A., Mäki A., van Elsas J.D., Nissinen
R. Plants assemble species specific bacterial communities from
common core taxa in three arcto-alpine climate zones. Front. Microbiol.
2017;8:12. DOI 10.3389/fmicb.2017.00012.

Kuzyakov Y., Blagodatskaya E. Microbial hotspots and hot moments in
soil: concept & review. Soil Biol. Biochem. 2015;83:184-199. DOI
10.1016/j.soilbio.2015.01.025.

Latimer A.M. Species diversity. In: El-Shaarawi A.H., Piegorsch W.W.
(Eds.) Encyclopedia of Environmetrics. Wiley, 2012. DOI 10.1002/
9780470057339.vas046.pub2.

Liu L., Zhu K., Wurzburger N., Zhang J. Relationships between plant
diversity and soil microbial diversity vary across taxonomic groups
and spatial scales. Ecosphere. 2020;11(1):e02999. DOI 10.1002/
ecs2.2999.

Malard L.A., Pearce D.A. Microbial diversity and biogeography in Arctic
soils: microbial diversity and biogeography. Environ. Microbiol.
Rep. 2018;10:611-625. DOI 10.1111/1758-2229.12680.

Mansouri H., Petit A., Oger P., Dessaux Y. Engineered rhizosphere: the
trophic bias generated by opine-producing plants is independent of
the opine type, the soil origin, and the plant species. Appl. Environ.
Microbiol. 2002;68(5):2562-2566. DOI 10.1128/AEM.68.5.2562-
2566.2002.

Manzoni S., Schimel J.P., Porporato A. Responses of soil microbial
communities to water stress: results from a meta-analysis. Ecology.
2012;93(4):930-938. DOI 10.1890/11-0026.1.

Marinari S., Mancinelli R., Campiglia E., Grego S. Chemical and biological
indicators of soil quality in organic and conventional farming
systems in Central Italy. Ecol. Indic. 2006;6(4):701-711. DOI
10.1016/j.ecolind.2005.08.029.

Mayfield M.M., Bonser S.P., Morgan J.W., Aubin I., McNamara S.,
Vesk P.A. What does species richness tell us about functional trait
diversity? Predictions and evidence for responses of species and
functional trait diversity to land-use change. Glob. Ecol. Biogeogr.
2010;19(4):423-431. DOI 10.1111/j.1466-8238.2010.00532.x.

Monkai J., Goldberg S.D., Hyde K.D., Harrison R.D., Mortimer P.E.,
Xu J. Natural forests maintain a greater soil microbial diversity than
that in rubber plantations in Southwest China. Agric. Ecosyst. Environ.
2018;265:190-197. DOI 10.1016/j.agee.2018.06.009.

Nair D.N., Padmavathy S. Impact of endophytic microorganisms on
plants, environment and humans. Sci. World J. 2014;2014:250693.
DOI 10.1155/2014/250693.

Philippot L., Raaijmakers J.M., Lemanceau P., van der Putten W.H.
Going
back to the roots: the microbial ecology of the rhizosphere.
Nat. Rev. Microbiol. 2013;11:789-799. DOI 10.1038/nrmicro3109.

Pingel M., Reineke A., Leyer I. A 30-years vineyard trial: plant communities,
soil microbial communities and litter decomposition respond
more to soil treatment than to N fertilization. Agric. Ecosyst. Environ.
2019;272:114-125. DOI 10.1016/j.agee.2018.11.005.

Raaijmakers J.M., Paulitz T.C., Steinberg C., Alabouvette C., Moënne-
Loccoz Y. The rhizosphere: a playground and battlefield for soilborne
pathogens and beneficial microorganisms. Plant Soil. 2009;
321:341-361. DOI 10.1007/s11104-008-9568-6.

Ren C., Chen J., Deng J., Zhao F., Han X., Yang G., Tong X., Feng Y.,
Shelton S., Ren G. Response of microbial diversity to C:N:P stoichiometry
in fine root and microbial biomass following afforestation.
Biol. Fertil. Soils. 2017;53:457-468. DOI 10.1007/s00374-017-
1197-x.

Ren C., Wang T., Xu Y., Deng J., Zhao F., Yang G., Han X., Feng Y.,
Ren G. Differential soil microbial community responses to the linkage
of soil organic carbon fractions with respiration across landuse
changes. For. Ecol. Manag. 2018;409:170-178. DOI 10.1016/
j.foreco. 2017.11.011.

Rosier A., Medeiros F.H.V., Bais H.P. Defining plant growth promoting
rhizobacteria molecular and biochemical networks in beneficial
plant-microbe interactions. Plant Soil. 2018;428:35-55. DOI
10.1007/s11104-018-3679-5.

Rousk J., Bengtson P. Microbial regulation of global biogeochemical
cycles. Front. Microbiol. 2014;5:103. DOI 10.3389/fmicb.2014.
00103.

Rousk J., Brookes P.C., Bååth E. Fungal and bacterial growth responses
to N fertilization and pH in the 150-year ‘Park Grass’ UK grassland
experiment: N and pH influence on microbial growth in grassland soils. FEMS Microbiol. Ecol. 2011;76(1):89-99. DOI 10.1111/
j.1574-6941.2010.01032.x.

Sally E.S., David R. Mycorrhizal Symbiosis. Elsevier, 2008. DOI
10.1016/B978-0-12-370526-6.X5001-6.

Santonja M., Foucault Q., Rancon A., Gauquelin T., Fernandez C., Baldy
V., Mirleau P. Contrasting responses of bacterial and fungal communities
to plant litter diversity in a Mediterranean oak forest. Soil
Biol. Biochem. 2018;125:27-36. DOI 10.1016/j.soilbio.2018.06.020.

Scharf B.E., Hynes M.F., Alexandre G.M. Chemotaxis signaling systems
in model beneficial plant–bacteria associations. Plant Mol.
Biol. 2016;90:549-559. DOI 10.1007/s11103-016-0432-4.

Schlaeppi K., Dombrowski N., Oter R.G., Ver Loren van Themaat E.,
Schulze-Lefert P. Quantitative divergence of the bacterial root microbiota
in Arabidopsis thaliana relatives. Proc. Natl. Acad. Sci.
USA. 2014;111(2):585-592. DOI 10.1073/pnas.1321597111.

Schmid M.W., Hahl T., van Moorsel S.J., Wagg C., De Deyn G.B.,
Schmid B. Feedbacks of plant identity and diversity on the diversity
and community composition of rhizosphere microbiomes from
a long-term biodiversity experiment. Mol. Ecol. 2019;28:863-878.
DOI 10.1111/mec.14987.

Shekhar V., Stӧckle D., Thellmann M., Vermeer J.E.M. The role of
plant root systems in evolutionary adaptation. Curr. Top. Dev. Biol.
2019;131:55-80. DOI 10.1016/bs.ctdb.2018.11.011.

Shen C., Xiong J., Zhang H., Feng Y., Lin X., Li X., Liang W., Chu H.
Soil pH drives the spatial distribution of bacterial communities along
elevation on Changbai Mountain. Soil Biol. Biochem. 2013;57:204-
211. DOI 10.1016/j.soilbio.2012.07.013.

Sheng M., Chen X., Zhang X., Hamel C., Cui X., Chen J., Chen H.,
Tang M. Changes in arbuscular mycorrhizal fungal attributes along
a chronosequence of black locust (Robinia pseudoacacia) plantations
can be attributed to the plantation-induced variation in soil
properties. Sci. Total Environ. 2017;599-600:273-283. DOI 10.1016/
j.scitotenv.2017.04.199.

Talbot J.M., Bruns T.D., Taylor J.W., Smith D.P., Branco S., Glassman
S.I., Erlandson S., Vilgalys R., Liao H.-L., Smith M.E.,
Peay K.G. Endemism and functional convergence across the North
American soil mycobiome. Proc. Natl. Acad. Sci. USA. 2014;111:
6341-6346. DOI 10.1073/pnas.1402584111.

Thoms C., Gattinger A., Jacob M., Thomas F.M., Gleixner G. Direct
and indirect effects of tree diversity drive soil microbial diversity in
temperate deciduous forest. Soil Biol. Biochem. 2010;42(9):1558-
1565. DOI 10.1016/j.soilbio.2010.05.030.

Trivedi P., Anderson I.C., Singh B.K. Microbial modulators of soil
carbon storage: integrating genomic and metabolic knowledge for
global prediction. Trends Microbiol. 2013;21(12):641-651. DOI
10.1016/j.tim.2013.09.005.

Tu Q., Deng Y., Yan Q., Shen L., Lin L., He Z., Wu L., Van Nostrand
J.D., Buzzard V., Michaletz S.T., Enquist B.J., Weiser M.D.,
Kaspari M., Waide R.B., Brown J.H., Zhou J. Biogeographic patterns
of soil diazotrophic communities across six forests in the
North America. Mol. Ecol. 2016;25(12):2937-2948. DOI 10.1111/
mec.13651.

van der Wal A., van Veen J.A., Smant W., Boschker H.T.S., Bloem J.,
Kardol P., van der Putten W.H., de Boer W. Fungal biomass development
in a chronosequence of land abandonment. Soil Biol. Biochem.
2006;38(1):51-60. DOI 10.1016/j.soilbio.2005.04.017.

Vandenkoornhuyse P., Quaiser A., Duhamel M., Le Van A., Dufresne A.
The importance of the microbiome of the plant holobiont. New Phytol.
2015;206(4):1196-1206. DOI 10.1111/nph.13312.

Wallander H., Ekblad A., Godbold D.L., Johnson D., Bahr A., Baldrian
P., Björk R.G., Kieliszewska-Rokicka B., Kjøller R., Kraigher H.,
Plassard C., Rudawska M. Evaluation of methods to estimate production,
biomass and turnover of ectomycorrhizal mycelium in forests
soils – a review. Soil Biol. Biochem. 2013;57:1034-1047. DOI
10.1016/j.soilbio.2012.08.027.

Walters K.E., Martiny J.B.H. Alpha-, beta-, and gamma-diversity of
bacteria varies across habitats. PLoS One. 2020;15:e0233872. DOI
10.1371/journal.pone.0233872.

Wang H.H., Chu H.L., Dou Q., Xie Q.Z., Tang M., Sung C.K.,
Wang C.Y. Phosphorus and nitrogen drive the seasonal dynamics
of bacterial communities in pinus forest rhizospheric soil of the
Qinling Mountains. Front. Microbiol. 2018;9:1930. DOI 10.3389/
fmicb.2018.01930.

Whitman W.B., Coleman D.C., Wiebe W.J. Prokaryotes: the unseen
majority. Proc. Natl. Acad. Sci. USA. 1998;95(12):6578-6583. DOI
10.1073/pnas.95.12.6578.

Whittaker R.H. Evolution and measurement of species diversity. Taxon.
1972;21:213-251. DOI 10.2307/1218190.

Xi N., Chu C., Bloor J.M.G. Plant drought resistance is mediated by
soil microbial community structure and soil-plant feedbacks in a
savanna tree species. Environ. Exp. Bot. 2018;155:695-701. DOI
10.1016/j.envexpbot.2018.08.013.

Xu Z., Yu G., Zhang X., He N., Wang Q., Wang S., Xu X., Wang R.,
Zhao N. Biogeographical patterns of soil microbial community as
influenced by soil characteristics and climate across Chinese forest
biomes. Appl. Soil Ecol. 2018;124:298-305. DOI 10.1016/j.apsoil.
2017.11.019.

Yang N., Ji L., Salahuddin Y., Yang L. The influence of tree species on
soil properties and microbial communities following afforestation of
abandoned land in northeast China. Eur. J. Soil Biol. 2018;85:73-78.
DOI 10.1016/j.ejsobi.2018.01.00.3.

Yao X., Zhang N., Zeng H., Wang W. Effects of soil depth and plant–
soil interaction on microbial community in temperate grasslands of
northern China. Sci. Total Environ. 2018;630:96-102. DOI 10.1016/
j.scitotenv.2018.02.155.

Zhalnina K., Dias R., de Quadros P.D., Davis-Richardson A., Camargo
F.A.O., Clark I.M., McGrath S.P., Hirsch P.R., Triplett E.W.
Soil pH determines microbial diversity and composition in the park
grass experiment. Microb. Ecol. 2015;69(2):395-406. DOI 10.1007/
s00248-014-0530-2.

Zverev A.O., Pershina E.V., Provorov N.A., Andronov E.E., Erikova
E.N. Metagenomic characteristic of rhizosphere effect on cereals
in black and sod-podzolic soils. Agric. Biol. 2016;51(5):654-663.
DOI 10.15389/agrobiology.2016.5.654eng.

